# Chronic sleep restriction during juvenility alters hedonic and anxiety-like behaviours in a sex-dependent fashion in adolescent *Wistar* rats

**DOI:** 10.3389/fnins.2024.1452429

**Published:** 2024-08-12

**Authors:** Anna Carolina Muniz Barreto, Julia Naomi Sakamoto Oliveira, Deborah Suchecki

**Affiliations:** Group of Studies on the Neurobiology of Stress and its Disorders – GENED, Department of Psychobiology – Escola Paulista de Medicina, Universidade Federal de São Paulo, São Paulo, Brazil

**Keywords:** sleep restriction, stress, adolescence, sex differences, sucrose splash test, anxiety-like behaviour, coping behaviour, corticosterone

## Abstract

Chronic reduction of sleep time in children and adolescents has been related to increased incidence of anxiety and depression. In rats, protocols of protracted sleep deprivation or chronic sleep restriction (CSR) are considered a stressor. In previous studies we showed that post-weaning CSR in male rats induces anxiety-like behaviour and changes in neurotransmission in emotion-related brain areas. In the present study we examined whether the effects of this adversity are sex-dependent. Twenty-two litters, containing four males and four females were distributed into control (CTL) and CSR groups. CSR began on postnatal day (PND) 21 and lasted for 21 days; each day the animals were placed onto small platforms immersed in water for 18 h and were allowed to sleep freely in their home-cages for the remaining 6 h. Throughout the CSR, all animals underwent the sucrose splash test once/week to assess their self-care and hedonic behaviours. Body weight was measured on PNDs 21 and 42. At the end of CSR period, the adolescents were allowed to sleep freely for 2 days, after which, behavioural tests began. Within each litter, one male and one female (pair) were not tested and provided blood and brain for determination of basal corticosterone (CORT) levels and hippocampal BDNF. One pair was tested in the sucrose preference test (SPT), one pair on the elevated plus maze (EPM) and one pair in the forced swim test (FST). CORT was measured after all conditions. CSR impaired self-care behaviour and body weight gain in males and females and increased relative adrenal weight only in males. There were no changes in sucrose intake in the SPT; CSR females displayed less immobility in the FST and CSR males displayed more anxiety-like behaviour in the EPM. CORT levels were similar between CTL and CSR males, whilst lower in CSR females than CTL ones in all experimental conditions. No changes in BDNF levels were detected in the dorsal hippocampus of CSR rats. The results indicate that CSR impaired self-care behaviour in both sexes, but only males displayed anxiety-like behaviour, whilst sleep recovery in females appeared to normalise their behaviour.

## 1 Introduction

The American Academy of Sleep Medicine (AASM) recommends that 13–18 year-old adolescents sleep 8 to 10 h/day on a regular basis to maintain physical and mental health (Paruthi et al., 2016). The circadian rhythm of the sleep–wake cycle changes throughout life and adolescents show a phase delay, i.e., they prefer to sleep and wake later than children and adults (Carskadon et al., 1997). In addition, REM sleep time increases at this age range (Tarokh et al., 2011). These physiological changes in sleep may lead to a significant reduction of sleep time due to environmental factors, such as longer exposure to artificial light, early school times, increased schoolwork, and social demands (Hysing et al., 2013; Chen et al., 2014). Currently, it is estimated that children and adolescents sleep 1 to 1.5 h less than recommended by the AASM (Matricciani et al., 2012), with a major negative impact on emotional regulation (Baum et al., 2014), metabolism (Simon et al., 2021), learning and memory (O'Brien, 2009; Short and Chee, 2019). Considering the intense morpho-functional brain changes characteristic of adolescence (Casey et al., 2015), this is a period of particular biopsychosocial vulnerability to stressful events (Casey et al., 2010). Human (Leproult et al., 1997; Balbo et al., 2010; Leproult and Van Cauter, 2010) and animal studies (Meerlo et al., 2002; Koban et al., 2006; Galvão et al., 2009; Martins et al., 2010; Moraes et al., 2022) show that sleep restriction and sleep deprivation activate the hypothalamic–pituitary–adrenal (HPA) axis at all levels, indicating that these are stressful situations. Although the activation of the HPA axis is increased by sleep deprivation or sleep restriction, the deactivation (negative feedback mechanism) does not appear to be affected (Suchecki et al., 2002a,b; da Silva et al., 2018).

Epidemiological studies show that chronic sleep restriction increases the risk for anxiety and depressive symptoms in adolescents (Zhang et al., 2017) and that social jetlag, e.g., the difference between the average sleep time during weekdays and weekend, higher than 1 h is a risk factor for irritable mood, increased daytime sleepiness and poor school performance (Tamura et al., 2022). Moreover, a prospective study showed that sleeping less than 6 h/night in 11- to 17-year-olds predicts depressive symptoms and major depression, assessed 1 year later (Roberts and Duong, 2014). However, anxiety and depression are known to impair sleep quality, increase sleep fragmentation and alter sleep architecture (Fang et al., 2019; Riemann et al., 2020; Chellappa and Aeschbach, 2022), indicating a bidirectional relationship between sleep and mental health (Chellappa and Aeschbach, 2022). Of particular importance is the evidence from epidemiological studies that anxiety and depression are more prevalent in women than in men and that the onset of this sex difference begins in adolescence (Kessler et al., 1993). Likewise, women are also more frequently plagued by insomnia than men (Theorell-Haglöw et al., 2018; Pajėdienė et al., 2024) and this sex difference emerges in adolescence (Zhang et al., 2016).

Despite the evidence of a possible relationship between reduced sleep time and affective behaviours in humans, animal models are useful to establish cause-effect relationships and disclose potential mechanisms under controlled conditions. In previous studies, our group showed that male *Wistar* rats submitted to chronic sleep restriction (CSR) for 21 days, from juvenility to mid adolescence, displayed higher levels of anxiety-like behaviour in the elevated plus maze (EPM) and dysregulation of monoaminergic neurotransmission in the limbic system (da Silva et al., 2018), which persisted until adulthood (Simionato et al., 2022). In addition, we also showed that CSR reduced BDNF levels in the dorsal hippocampus, measured after the behavioural tests (da Silva et al., 2018). These results suggest that CSR during this neurodevelopmental period may permanently alter behavioural regulation and the corresponding neurobiology in male rats.

Given that in humans sex differences in prevalence of insomnia, anxiety and mood disorders emerge during adolescence, in the present study we tested whether CSR during juvenility and early adolescence would lead to greater impact on affective behaviour and stress response in female than in male rats. To test this hypothesis, mid-adolescent rats from both sexes were assessed after CSR in tests related to anxiety-, depressive- and coping-like behaviours, basal and post-test corticosterone was determined in plasma. BDNF levels were determined in the dorsal hippocampus in non-tested rats, to examine whether CSR alone would impact this neurotrophin. We also assessed the phase of the oestrous cycle on the euthanasia day to determine whether eventual behavioural changes in females could be explained by this parameter.

## 2 Materials and methods

### 2.1 Animals

Twenty-two litters were obtained from the breeding *Wistar* rats, in a 2:1 female to male ratio. Animals were acquired from Centro de Desenvolvimento de Modelos Experimentais para Medicina e Biologia (CEDEME) from Universidade Federal de São Paulo. The day of birth was designated as postnatal day (PND) 0 and litters were culled to four males and four females on postnatal day (PND) 1. All protocols and procedures were approved by the local Ethics Committee (CEUA process numbers 9,462,090,218 and 859,090,218). The animals were housed in polypropylene cages (30 cm x 38 cm x 15 cm) under controlled temperature (22 ± 2°C) and lighting conditions (12 h light/dark cycle, starting at 7:00 a.m.—*zeitgeber* time [ZT] 0). Pups were weaned on PND 21 and same-sex siblings were housed together.

### 2.2 Chronic sleep restriction

This protocol involves an 18 h period of sleep deprivation (from ZT 9 to ZT 3 on the next day) and 6 h (from ZT 3 to ZT 9) of free sleep in the home cage (Machado et al., 2005). Sleep deprivation was accomplished by placing 4 same-sex pups on water containers, containing twice as many narrow platforms (Suchecki and Tufik, 2000). On PND 18, half of the number of litters were designated to the control group (CTL) and were kept in their home-cages during the entire protocol period, whereas the other half was exposed to the CSR protocol. Briefly, on PND 18 the pups were habituated to 3 cm platforms, immersed in water up to 1 cm below the upper surface, for 1 h/day, for 3 days. At weaning, on PND 21 the CSR began, with small adjustments on the 1st week, due to their age and size, so the sleep restriction would not be so extreme and the proportion between body size and platform diameter would be preserved. Between PND 21 and 27, the platform size and duration of sleep deprivation was increased incrementally from 3.0 cm for 14 h/day to 4.5 cm for 16 h/day. On PND 28, they were placed on 6.5 cm platforms for 18 h/day, for 14 days (da Silva et al., 2018). After the end of the protocol, on PND 42, the animals were allowed to sleep freely for 48 h, after which the behavioural tests began (on PND 44).

### 2.3 Behavioural tests

Within each litter one male and one female were blood sampled without being exposed to any test—non-tested (NT), one male and one female were submitted to the sucrose preference test (SPT), one male and one female were tested in the elevated plus maze (EPM), and one male and one female were submitted to the forced swim test (FST). We did this to keep the same interval between the CSR and the onset of behavioural tests. The total number of animals was reached in three replications.

#### 2.3.1 Sucrose splash test

The SST was carried out with all animals. The first test took place immediately before the onset of sleep restriction (baseline) and afterwards, once a week during the CSR period (PND 28 [T1], PND 35 [T2] and PND 42 [T3]), always at ZT 8. This test assesses self-care and hedonic behaviours (Machado et al., 2012b) and the protocol was adapted from Ducottet et al. (2004), to be used in rats. Each animal was housed in a cage for 30 min, after which they received two squirts of a 10% sucrose solution on the back. The solution dirties the animal’s coat triggering grooming behaviour. The test was videotaped for 10 min and the latency to initiate grooming and the frequency of facial and body grooming were recorded. Depressive-like behaviour is characterised by increased latency to and decreased frequency of grooming.

For this behaviour, 66 rats of both sexes (Females: CTL = 16; CSR = 17; Males: CTL = 16; CSR = 17), were submitted to the SST; there are missing data due to a power failure in the animal facility on the day that one of the SST should have taken place.

#### 2.3.2 Sucrose preference test

On PND 44, at ZT 4, 39 animals were submitted to the SPT (Females: CTL = 10; CSR = 10; Males: CTL = 9; CSR = 10) by using the sucrose positive contrast test, which measures the ability of rats to detect a change in the sucrose solution palatability (Matthews et al., 1996). The animals were individually housed for 3 days, during which two bottles containing a sucrose solution or water, were offered. On the first 2 days one bottle contained a 1% sucrose solution (200 mL) and on the third day the bottle contained a 10% sucrose solution. Each day, the bottles were weighed before and after 24 h of free intake and the sucrose preference index was calculated by dividing the weight difference in the sucrose bottle by the sum of weight difference of both bottles. To prevent location bias, the position of the bottles was switched every day.

#### 2.3.3 Elevated plus maze

Thirty-one rats (Females: CTL = 8; CSR = 8; Males: CTL = 7; CSR = 8) were tested in the EPM on PND 44, between ZT 2 and ZT 5, under dim light (16 lux). The apparatus is made of wood, located 50 cm above the floor, and consisted of two open arms (50 cm long and 10 cm wide each), perpendicular to two arms of the same size, closed by 40-cm high walls and a central open segment (10 × 10 cm) common to both arms. Each arm is divided into three segments (16 × 10 cm), which are useful to record the magnitude of exploration by the animal. The 5-min long test was video-recorded for offline analysis. Each animal was placed in the central segment facing one open arm and left to freely explore the new environment. The parameters analysed were (1) percentage of entries in the open arms (%EOA), calculated by dividing the number of entries in the open arms (OA) by the number of entries in all arms; (2) percentage of time in the open arms (%TOA), calculated by dividing the time spent in the OA by 600 (length of the test in seconds); (3) percentage of segments crossed in the OA (%SOA), calculated by dividing the number of segments crossed in the OA by the total number of segments crossed. The Anxiety Index (AI) reflects the level of general open arms exploration and is calculated by the equation AI = 100–[(%TOA) + (%EOA)]/2 (Mazor et al., 2009).

#### 2.3.4 Forced swim test

The FST was used to assess stress coping behaviour under an inescapable situation (Molendijk and de Kloet, 2015; de Kloet and Molendijk, 2016). In humans, passive stress coping style predicts symptoms of depression (Nagase et al., 2009). For this test, 9 CTL and 10 CSR females and 9 CTL and 9 CSR males were individually trained for 15 min, between ZT 2 and ZT 5, in a water-filled acrylic container (30 cm in diameter x 50 cm in height, water at 25 ± 1°C and up to 30 cm); 24 h later (PND 45), they were put back in the water container for a 5-min test, which was video-recorded for offline behavioural analysis. The behaviour expressed, swimming, climbing or immobility, was assessed every 5 s and recorded (mounting up to 60 observations), in addition to the latency to immobility (Hamani et al., 2012).

### 2.4 Sampling

Thirty min after the end of each test or at the corresponding time-point for NT counterparts, the animals were decapitated, and trunk blood was collected in chilled vials containing 1 drop of 7.5% EDTA solution. Blood sampling was done without anaesthesia, for it interferes with corticosterone (CORT) levels (Bhathena, 1992; Vahl et al., 2005; Arnold and Langhans, 2010). The brains were harvested, immediately frozen and kept at −80°C until determination of dorsal hippocampus BDNF levels.

### 2.5 Body weight gain

Body weight was determined on PNDs 21 (before the beginning of the protocol) and 42, in females (CTL = 40 and CSR = 32) and males (CTL = 30; CSR = 32) and the weight variation between the two ages was used as a measure of body weight gain.

### 2.6 Relative adrenal weight

Adrenal glands were excised, cleaned of the surrounding fat and weighed in analytical scale (Females: CTL = 40; CSR = 36; Males: CTL = 37; CSR = 34). The relative adrenal weight was calculated by the formula: [(weight of both adrenals (mg)/body weight (g)) x 1,000]. The body weight used for this calculation was obtained on the day of euthanasia. Augmented adrenal relative weight is used as an index of chronic stress (Sapolsky et al., 2000).

### 2.7 Corticosterone plasma levels

Blood samples (Females: CTL = 37; CSR = 33; Males: CTL = 33; CSR = 35) were collected and centrifuged for 15 min at 2300 RPM for 20 min at 4°C and the plasma was separated and kept frozen at – 20°C until the assay. The method used for CORT determination was described by Lopez-Jiménez and coworkers (Lopez-Jimenez et al., 1989). Briefly, plasma aliquots (25 μL) were resuspended 1 mL of ethanol for steroid extraction and centrifuged. The supernatant was transferred to another tube and vacuum-lyophilized at 35°C for 90 min. Subsequently, the samples were resuspended in 2.5 mL 1% gel buffer. The samples were pipetted (0.5 mL), in duplicate, and 0.1 mL of rabbit anti-corticosterone (Sigma C8784), together with 0.1 mL of [^3^H]-corticosterone (Perkin-Elmer) were added to each tube and maintained at 4°C for 15 h. Afterwards, 0.2 mL of 0.5/0.005% charcoal/dextran solution and the tubes were centrifuged at 2500 for 15 min at 4°C, The supernatant was transferred to tubes and 4 mL of scintillation liquid was added and the tubes were left in the dark for 2 h. The radioactivity was determined by a beta-counter (LS 6500 – Beckman, Beckman Instruments Inc., Fullerton, CA, United States).

### 2.8 Determination of the phase of oestrous cycle

Immediately after decapitation, vaginal smears were collected from 43 CTL and 44 CSR female rats. The material was spread in glass slides, stained by the Shorr method and observed in optic microscope (Paccola et al., 2008). The phase of oestrous cycle (proestrus, oestrous, metestrus or dioestrus) was defined according to the histological characteristics in the smear (Goldman et al., 2007). The evaluation of the phase was performed by A.C.M.B for all females.

### 2.9 BDNF levels in the dorsal hippocampus

BDNF levels were determined in dorsal hippocampus of non-tested (NT) males (CTL = 6; CSR = 10) and females (CTL = 8; CSR = 10). The hippocampi were dissected in a cold surface. The tissue was weighed and transferred to a vial containing 300 μL of lysis buffer/5 mg of tissue, following the company’s protocol (Abcam, Western blotting—a beginner’s guide) for the preparation of homogenates. Each vial was centrifuged for 20 min at 12,000 rpm at 4°C and the supernatant was used for BDNF determination. Protein levels in each sample were determined by Lowry method with Bio-Rad commercial kit (DC Protein Assay, albumin standard 500–0112, reagents 500–0116). BDNF levels were determined by using a commercial ELISA kit from BT-LAB (Zhejiang, China).

### 2.10 Experimental design

**Figure 1:**
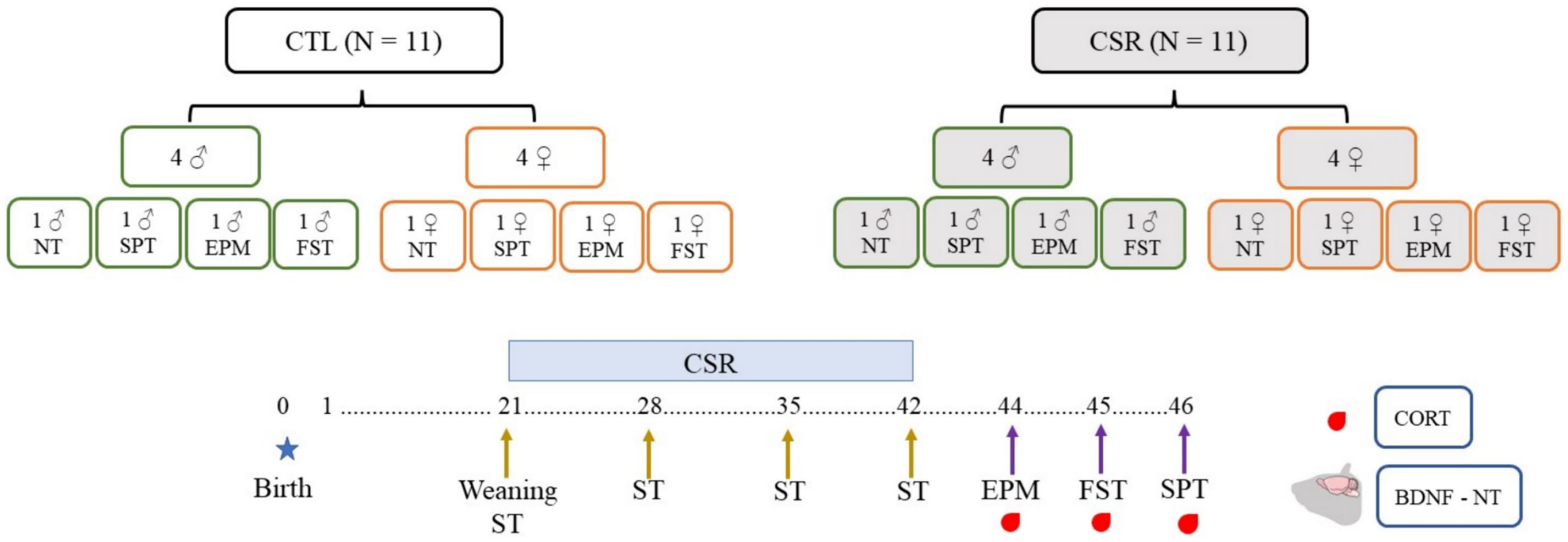
Experimental design of the present study. Litters were distributed in control (CTL, white boxes) or chronic sleep restriction (CSR, grey boxes). Litters were standardised to four males (green contour) and four females (orange contour), and, after the CSR period, distributed into four conditions: non-tested (NT) or exposed to the sucrose preference test (SPT), to the elevated plus maze (EPM) or to the forced swim test (FST). Throughout the CSR or for CTL animals, the sucrose splash test (SST) was performed once a week.

Litters composed of four males and four females were designated to the CTL or CSR groups. Three days before weaning (PND 18), litters of the CSR group were habituated to the platforms over water for 1 h/day. On PND 21 the juveniles were weighed and placed onto the platforms at 4 p.m. until 10 a.m. the next day, when they were transferred to their home-cages (same-sex siblings). This procedure was repeated every day, for 21 days. Immediately before the beginning and once/week during the CSR, we performed the SST. After the last day of sleep deprivation, animals were weighed again, returned to their home cages and allowed to sleep freely for 48 h. After this recovery period they were distributed into 4 conditions: non-treated (NT), sucrose preference test (SPT), elevated plus maze (EPM) and forced swim test (FST). Thirty min after the end of each test or at the corresponding time for NT rats (animals were distributed to match blood sampling of tested counterparts), rats were blood sampled, adrenals and brains were harvested. Adrenals were immediately weighed, and brains were washed in 0.9% saline and frozen at −80°C (Figure 1).

### 2.11 Statistical analysis

Data analysis was carried out with Statistica software v.14 (StatSoft Inc., 2013).^1^ Normality and homogeneity of data distribution were determined by Shapiro–Wilk and Levene tests and parameters were analysed by General Linear Models (GLM), separately for sex. Variables of the SST and SPT were analysed by a two way-ANOVA for repeated measures (Group [CTL, CSR] x Time-point [SST: Basal, Week 1, Week 2, Week 3; SPT: Day 1, Day 2, Day 3]). Corticosterone levels were analysed by a 2-way factorial ANOVA with Group and Condition (non-tested [NT], post-SPCT, post-EPM, post-FST). All other variables were analysed by between groups comparison. When appropriate, Newman–Keuls *post-hoc* test was employed to detect pairwise differences. In all cases, the statistical significance was considered when *p* ≤ 0.05.

The GLM provides information of effect size (eta-square, η^2^) for each factor and the interaction between factors. These indices are interpreted as small effect 0.01 to 0.059; moderate effect 0.06 to 0.139; and high effect 0.14 and above (Statology, 2021; Glen, 2022).

## 3 Results

A total of 46 CTL and 43 CSR females, 40 CTL and 43 males were obtained from 11 litters per group. However, due to attrition caused by technical problems, the number of animals evaluated in the different tests is different from the total and was specified in the Methods session and in the figure captions.

### 3.1 Sucrose splash test

**Figure 2:**
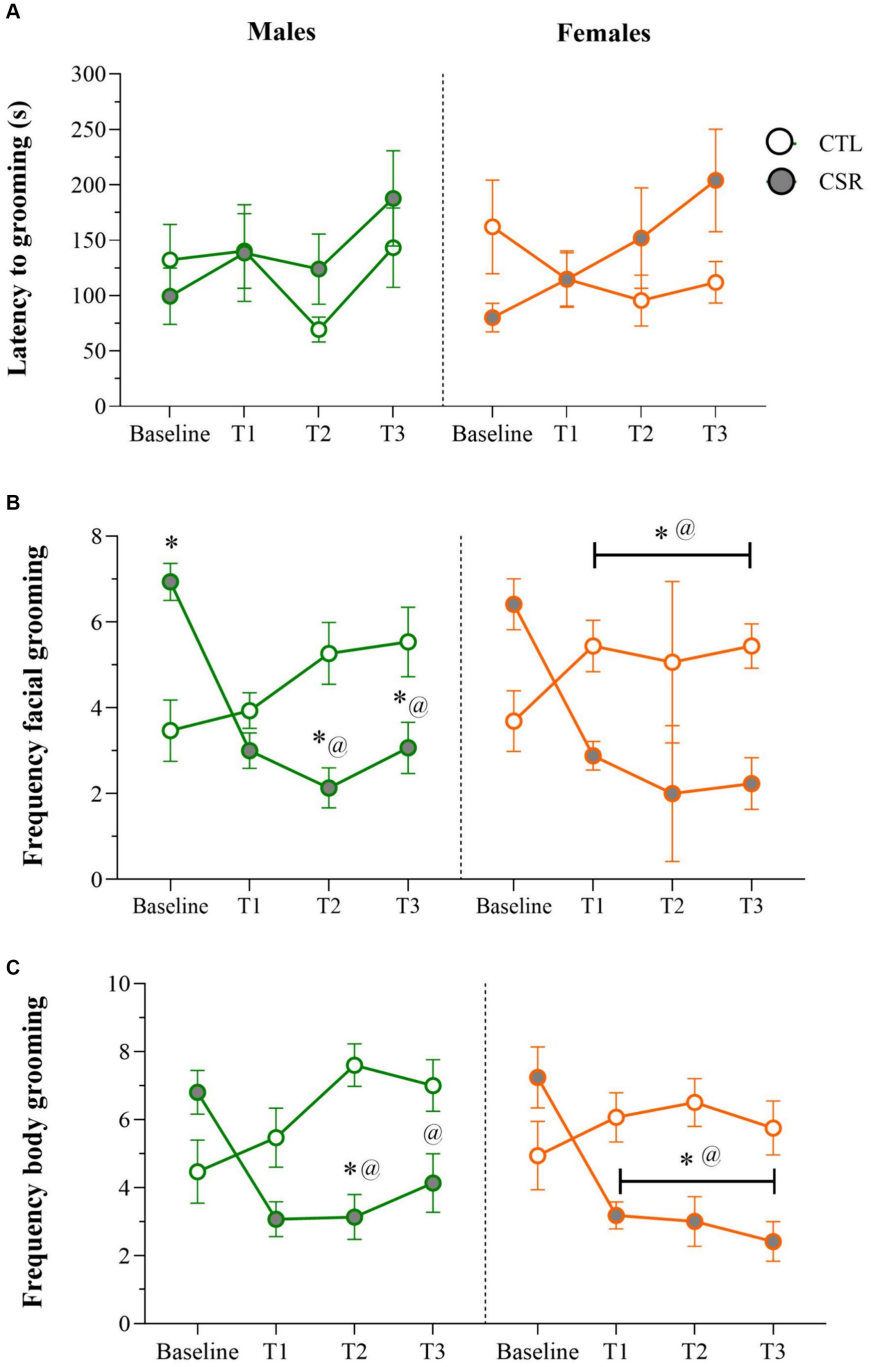
Parameters of the Sucrose Splash Test (SST) during the sleep restriction period, in male (green) and female (orange) adolescents, submitted to chronic sleep deprivation (CSR) or not (control – CTL). **(A)** Latency to initiate grooming behaviour; **(B)** frequency of facial grooming; **(C)** frequency of body grooming. *difference from CTL group; @difference from baseline. The values are presented as the mean ± s.e.m. of CTL = 16 males and females; CSR = 17 males and females.

− Males: No differences were detected for the **onset** of grooming behaviour throughout the sleep restriction period. For **facial grooming**, there was a main effect of Time-point and an interaction between Group and Time-point [*F*_(3,84)_ = 12.709; *p* < 0.0001; η^2^ = 0.31]. Analysis of the interaction revealed that CTL and CSR groups were different at baseline, weeks 2 and 3 (p’s < 0.05). For the CSR group there was a reduction of facial grooming from week 1 on, compared to baseline (p’s < 0.0005). As for **body grooming**, there was a main effect of Group and an interaction between Group and Time-point [F_(3,84)_ = 6.413; *p* < 0.001; η^2^ = 0.186]. The Newman–Keuls test showed that CSR group groomed less than CTL on week 2 (*p* < 0.03) and that CSR males groomed less on weeks 1 and 2 than baseline (*p* < 0.006).
− Females: A main effect of Time-point was detected for the **onset** of grooming [*F*_(3,96)_ = 3.275; *p* < 0.03; η^2^ = 0.09], but the *post hoc* test did not reveal further differences. Main effect of Group, and an interaction between Group and Time-point [F_(3,96)_ = 13.237; *p* < 0.0001; η^2^ = 0.29] was observed for frequency of **facial grooming**. Pairwise comparisons showed that CSR females displayed less grooming than CTL ones at all time-points (p’s < 0.02) and that CTL females displayed increased frequency throughout the time (p’s < 0.05), whereas CSR females showed the opposite profile (p’s < 0.002). Finally, analysis of **body grooming** detected main effects of Group and Time-point, and an interaction between both factors [F_(3,96)_ = 8.26; p < 0.001; η^2^ = 0.205]. Again, CSR females groomed less than CTL ones at all time-points, except from baseline (p’s < 0.03) and throughout the sleep restriction period, CSR females decreased the frequency of grooming, compared to baseline (p’s < 0.002) (Figure 2).

### 3.2 Sucrose preference test

**Figure 3:**
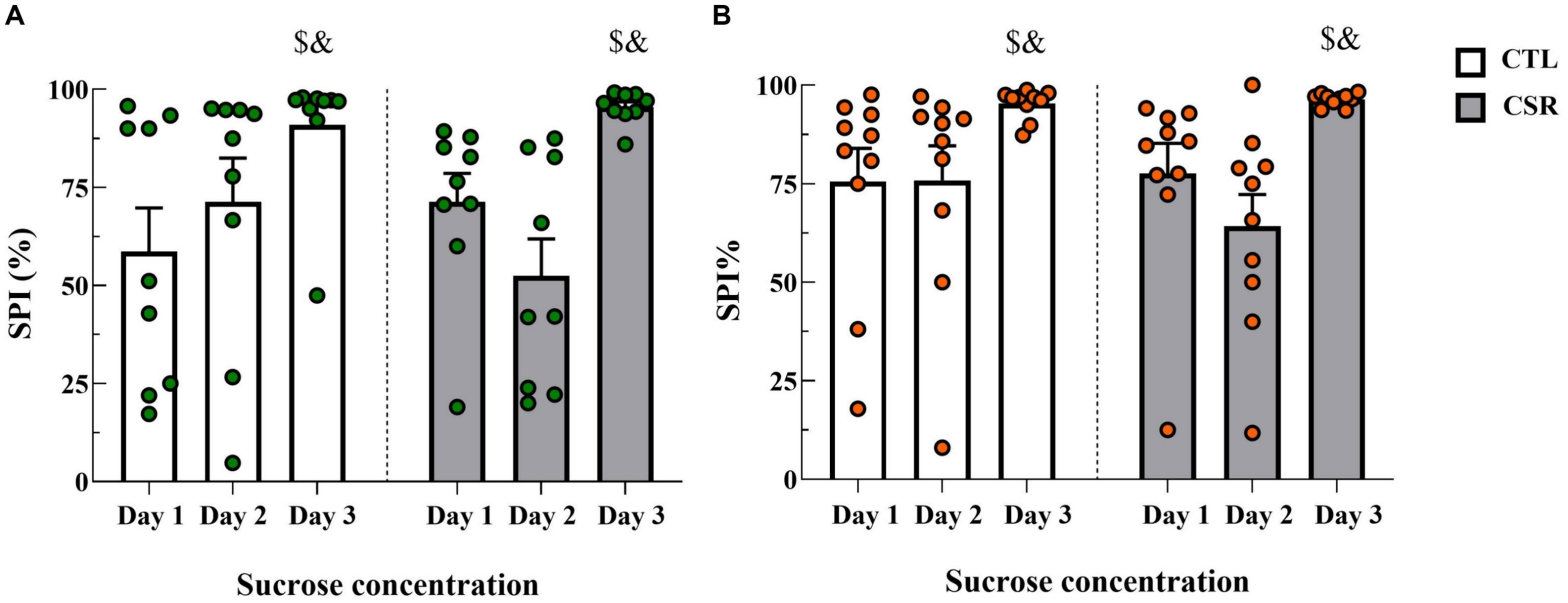
Sucrose preference index (SPI %) on each day of the SPT. White bars represent control (CTL) animals and grey ones, the chronic sleep restriction (CSR) group. Males **(A)** are represented by green circles and females **(B)** by orange circles. $different from Day 1; &different from Day 2. Individual values are shown, and the bars represent mean ± s.e.m. (*N* = Males: CTL = 9; CSR = 10; Females: CTL = 10; CSR = 10).

− Males: Analysis of sucrose preference index revealed a main effect of Time-Point [*F*_(2,32)_ = 14.025; *p* < 0.0001; η^2^ = 0.467] and a nearly significant interaction between Group and Time-Point [F_(2,32)_ = 3.168; *p* < 0.06; η^2^ = 0.165]. The *post hoc* analysis of the time-point indicated that sucrose preference was higher for the 10% solution (Day 3) than on the other days (*p* < 0.001) (Figure 3A).
− Females: There was a main effect of Time-Point [F_(2,32)_ = 10.052; *p* < 0.001; η^2^ = 0.358]. Like in males, intake of the concentrated sucrose solution was higher than intake of 1% solution (*p* < 0.03) (Figure 3B).

### 3.3 Elevated plus maze

**Figure 4:**
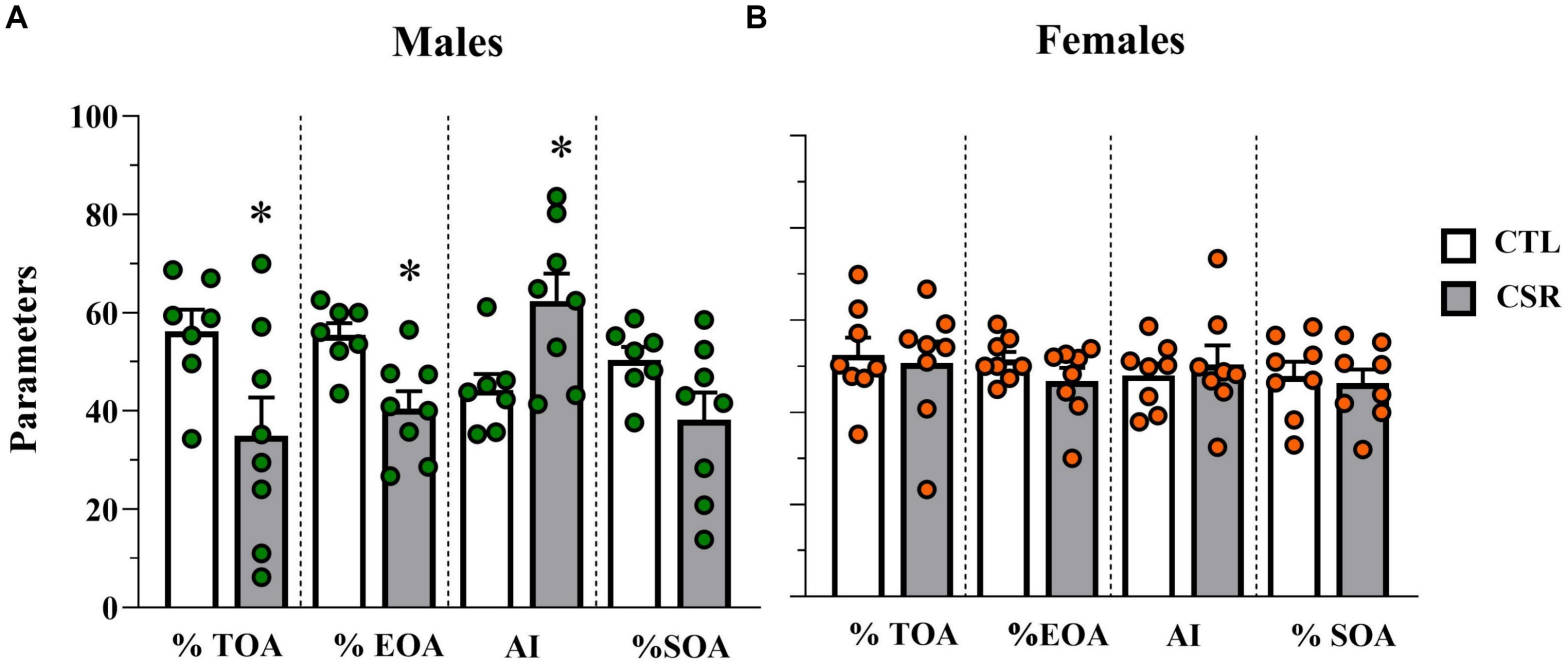
Parameters assessed in the elevated plus maze. White bars represent control (CTL) animals and grey ones, the chronic sleep restriction (CSR) group. Males **(A)** are represented by green circles and females **(B)** by orange circles. *difference from CTL group. Individual values are shown, and the bars represent mean ± s.e.m. (*N* = Males: CTL = 7; CSR = 8; Females: CTL = 8; CSR = 8). %TOA, percentage of time in the open arms; %EOA, percentage of entries in the open arms; AI, anxiety index; %SOA, percentage of squares crossed in the open arms.

− Males: CSR reduced %TOA [*F*_(1,13)_ = 5.2; *p* < 0.004; η^2^ = 0.286], %EOA [F_(1,13)_ = 11.321; *p* < 0.005; η^2^ = 0.465], leading to greater Anxiety Index [F_(1,13)_ = 7.295; *p* < 0.02; η^2^ = 0.36]. Animals in the CSR group also reached the farther segment of the open arms less than the CTL group [F_(1,13)_ = 4.7; *p* < 0.05; η^2^ = 0.266. CTL = 8.57 ± 2.0; CSR = 5.62 ± 3.01]. No group differences were observed for the number of squares crossed in the open arms (Figure 4A).
− Females: There were no group differences in any of the parameters analysed (%TOA [*F*_(1,14)_ = 0.0; *p* < 1.0]; %EOA [F_(1,14)_ = 2.014; *p* < 0.18; η^2^ = 0.12]; AI [F_(1,14)_ = 0.225; *p* < 0.64; η^2^ = 0.016], %SOA [F_(1,14)_ = 0.134; *p* < 0.72; η^2^ = 0.009]; extremity of OA [F_(1,13)_ = 0.097; *p* < 0.76; η^2^ = 0.007]) (Figure 4B).

### 3.4 Forced swim test

**Figure 5:**
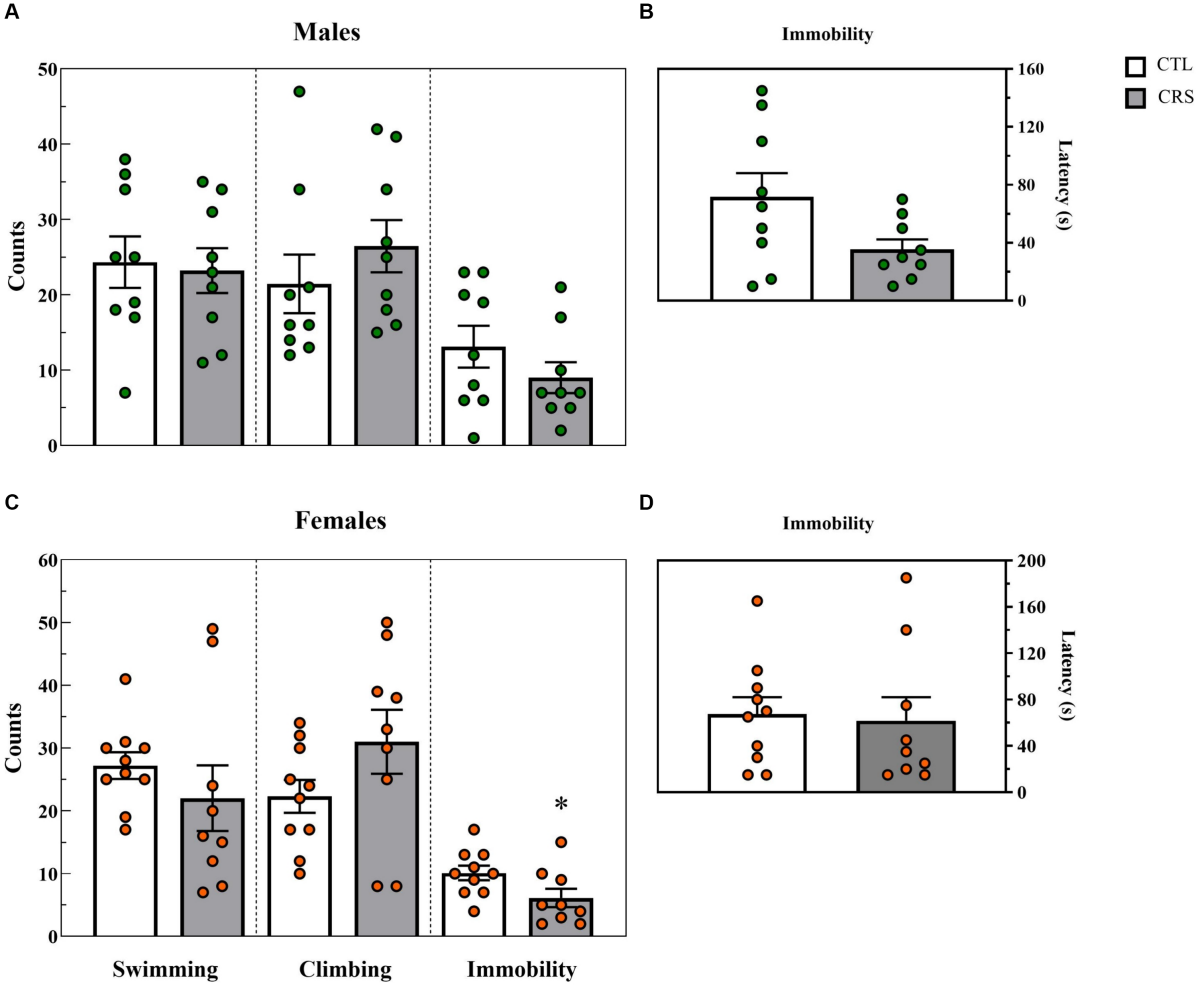
Parameters assessed in the forced swim test, counts of each behaviour **(A,C)** and latency (s) to immobility **(B,D)**. White bars represent control (CTL) animals and grey ones, the chronic sleep restriction (CSR) group. Males **(A,B)** are represented by green circles and females **(C,D)**, by orange circles. *difference from CTL group. Individual values are shown, and the bars represent mean ± s.e.m. (*N* = Males: CTL = 9; CSR = 9; Females: CTL = 9; CSR = 10).

− Males: A trend for reduced latency to immobility was observed in the CSR group [*F*_(1,16)_ = 4.132; *p* < 0.06; η^2^ = 0.205] (Figure 5B). No other differences were observed between groups (Swim: [F_(1,16)_ = 0.0602; *p* < 0.81; η^2^ = 0.004]; Climbing: [F_(1,16)_ = 0.92; *p* < 0.35; η^2^ = 0.05]; Immobility: [F_(1,16)_ = 1.42; *p* < 0.25; η^2^ = 0.008]) (Figure 5A).
− Females: A significant difference between groups was observed for immobility counts [*F*_(1,17)_ = 4.646; *p* < 0.05; η^2^ = 0.215] and CSR females displayed less immobility than CTL ones (Figure 5C). No other differences were revealed (Latency to immobility: [F_(1,17)_ = 0.056; *p* < 0.82; η^2^ = 0.003]; Swim: [F_(1,17)_ = 0.92; *p* < 0.35; η^2^ = 0.05]; Climbing: [F_(1,17)_ = 2.46; *p* < 0.14; η^2^ = 0.13]).

### 3.5 Body weight and relative adrenal weight

**Figure 6:**
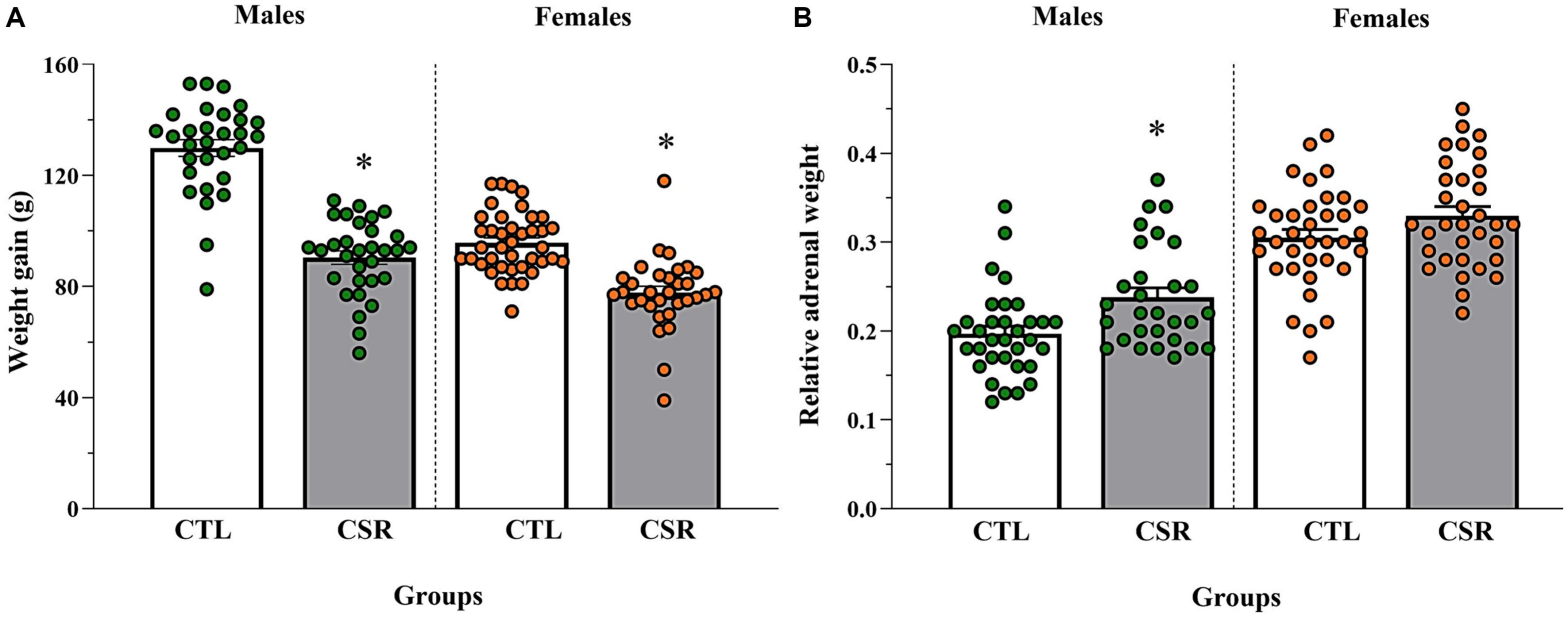
Body weight gain, measured by the variation between PND 21 and PND 42 **(A)** and adrenal weight relative to the body weight on euthanasia day **(B)**. The white bars represent the control (CTL) group and the grey ones, chronic sleep restriction (CSR) group. Males are represented by green and females, by orange circles. *different from CTL group. Individual values are shown, and the bars represent mean ± s.e.m. [N for **(A)**/**(B)** = Males: CTL = 30/33; CSR = 31/29; Females: CTL = 40/36; CSR = 32/33].

− Males: CSR animals showed smaller weight gain [*F*_(1,59)_ = 104.903; *p* < 0.00001; η^2^ = 0.64] and heavier adrenals [*F*_(1,60)_ = 9.793; *p* < 0.003; η^2^ = 0.14] than CTL counterparts.
− Females: CSR reduced body weight gain of females, compared to CTL animals [*F*_(1,70)_ = 41.158; *p* < 0.00001; η^2^ = 0.37], but there were no differences in the relative adrenal weight [*F*_(1,67)_ = 3.166; *p* < 0.08; η^2^ = 0.045]. Results are shown in Figure 6.

### 3.6 Corticosterone plasma levels

**Figure 7:**
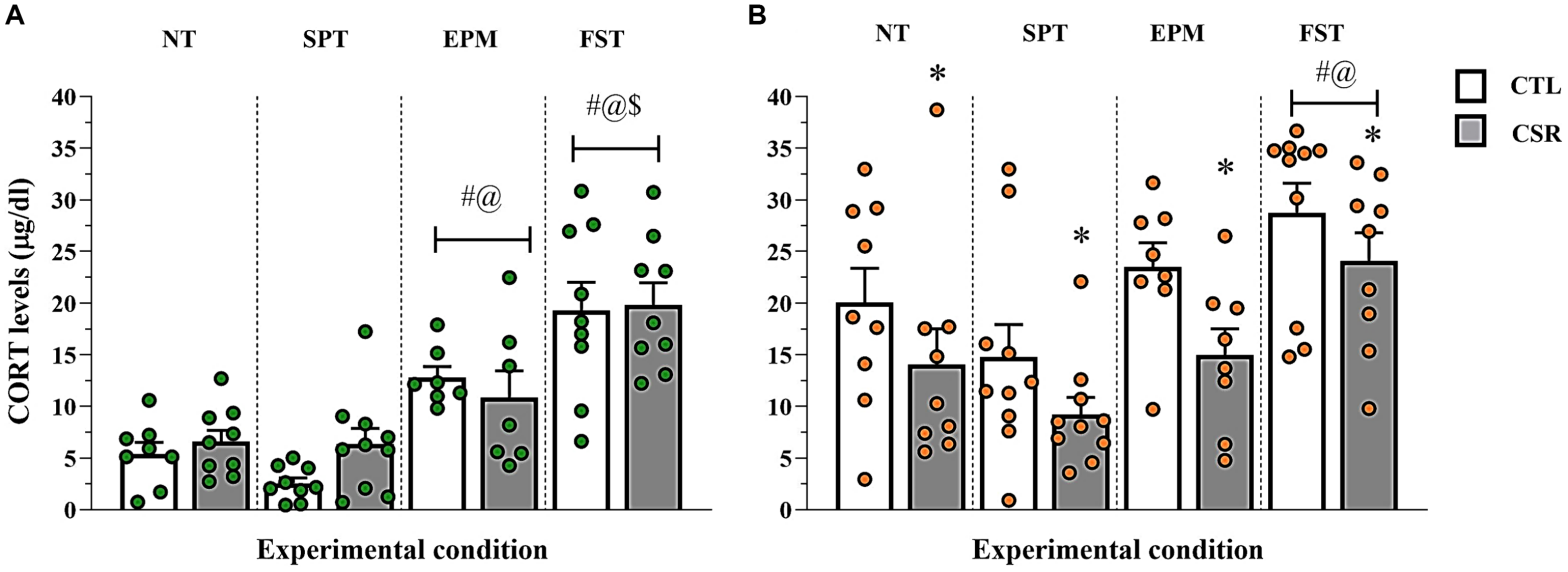
Plasma corticosterone (CORT) levels. The white bars represent the control (CTL) group and the grey ones, chronic sleep restriction (CSR) group. Males **(A)** are represented by green and females **(B)** by orange circles. Individual values are shown, and the bars represent mean ± s.e.m. *different from CTL group; #different from non-tested (NT) rats; @different from rats exposed to the sucrose preference test (SPT); $different from rats exposed to the elevated plus maze (EPM). FST, forced swim test.

− Males (Figure 7A): A main effect of Condition [*F*_(3,60)_ = 32.34; *p* < 0.0001; η^2^ = 0.62]. The *post-hoc* analysis showed that levels post-SPCT were similar to NT and both were lower than levels post-EPM (*p* < 0.002), which, in turn, were lower than post-FST (*p* < 0.005).
− Females (Figure 7B): Main effects of Group [*F*_(3,65)_ = 9.578; *p* < 0.003; η^2^ = 0.128] and Condition [F_(3,65)_ = 9.554; *p* < 0.0005; η^2^ = 0.306] were observed. In all conditions, CSR females secreted less CORT than CTL ones. CORT levels were the highest after FST (*p* < 0.02) and after SPCT, CORT levels were lower than after the EPM (*p* < 0.04).

### 3.7 Phase of oestrous cycle

Table 1 shows the distribution of females of each group in different phases of the oestrous cycle.

**Table 1:** Number of females of each group in different phases of the oestrous cycle.

	Phase of the oestrous cycle			
Group	Dioestrus	Oestrous	Metestrus	Proestrus
CTL	8	10	5	10
CSR	10	9	4	10

The phase was determined immediately after blood sampling.

### 3.8 Hippocampal BDNF levels

There were no effects of Group for males [*F*_(1,14)_ = 0.153; *p* < 0.24; η^2^ = 0.099] or for females [F_(1,16)_ = 0.001; *p* < 0.949; η^2^ = 0.0003]. The levels of BDNF in males were CTL = 86.8 ± 2.0 x CSR = 84.4 ± 4.2 pg./mg of tissue, and in females, CTL = 84.6 ± 3.2 x CSR = 84.6 ± 3.6 pg./mg of tissue.

## 4 Discussion

The results of the present study revealed an important sexually dimorphic effect of CSR on anxiety- and coping-like behaviours, in addition to the CORT response to the different behavioural tests. Interestingly, in both sexes CSR impaired self-care and hedonic behaviours measured by the SST, but after a two-day period of free sleep, CSR females behaved similarly to CTL ones in most tests, whereas males showed greater anxiety-like behaviour, replicating our previous study (da Silva et al., 2018).

The SST is a translational approach to assess depressive-like behaviour in rodents, because it simultaneously measures two behaviours that are evocative of core depressive symptoms, e.g., anhedonia and impairment of self-care (APA, 2013). Thus, when the highly palatable, 10% sucrose solution is squirted on the back of the animal, the viscosity incites body grooming and when the animal licks its paws for the first time, the hedonic value of the solution increases the frequency of facial grooming. Chronic stress has been shown to increase the latency to and to reduce the time spent grooming (Hu et al., 2017). Antidepressant drugs, such as fluoxetine and desipramine, on the other hand, increase grooming behaviour in the SST (Yalcin et al., 2005; Machado et al., 2012a). The reduction of body and facial grooming in CSR-exposed rats indicates that this condition precipitates depressive-like behaviour. This result is highly relevant to humans since CSR and the SST have a major translational value. Accordingly, there is evidence from an epidemiological study that reduced sleeping time increases the incidence of depressive symptoms in adolescents (Zhang et al., 2017). However, a recent systematic review and meta-analysis showed discrepant results about the relationship between sleep time and depressive symptoms in this population (Short et al., 2020).

After 48 h of sleep recovery animals were tested in the SPT, another test of anhedonic behaviour. In this test, a 1% sucrose solution was offered to the animals for two consecutive days and on the third day, a 10% solution. If CSR animals were anhedonic, they would not consume the 10% solution as much as CTL rats or no more than they consumed of the 1% solution (Matthews et al., 1996). In a previous study, we tested the animals in the negative contrast sucrose test, also after a 48-h sleep recovery. In this version of the test, animals need to realise that the sucrose solution on the 3^rd^ day is less palatable than in the previous 2 days (lowering from 15 to 5% concentration) and reduce their intake of the 5% solution (da Silva et al., 2018). No group differences were observed in either version of the test. Since CSR induced behavioural changes in the SST, but in the SPCT both groups behaved in a very similar way, that suggested that sleep recovery was sufficient to normalise depressive-like behaviour in both males and females.

In the EPM, CSR females behaved similarly to CTL ones, but males showed a clear increase in anxiety-like behaviour, with reduced exploration of the open arms, replicating our previous data (da Silva et al., 2018) and demonstrating a sex-dimorphic effect of CSR followed by a short sleep recovery period. Interestingly, in male rats exposed to the same protocol used in the present study, we showed that increased anxiety-like behaviour was still detected in the CSR group, even 45 days after the end of the protocol (Simionato et al., 2022). Of notice, there are very few animal studies on the immediate and long-term effect of CSR applied during development, and the species used, length and method of sleep deprivation/day and the duration of the protocols are different, generating a variation of results. For instance, 3 h of gentle handling between PNDs 5 and 42 failed to induce any behavioural alterations in the EPM in male or female mice tested 5 weeks later (Sare et al., 2016). On the other hand, 4 h of sleep deprivation by gentle handling/day for 8 days in 42-day-old mice decrease the latency to and increase the duration of immobility in the FST in females, but not in males (Murack et al., 2021). In the present study we found a trend with high effect size for shorter latency to immobility in CSR males and a significant decrease of immobility counts in CSR females.

The FST is a classical test for antidepressant drugs, for they reduce immobility time by increasing climbing (drugs that increase noradrenaline) and/or swimming (drugs that increase serotonin; Cryan et al., 2005). However, alternative interpretations for immobility on the FST have been proposed, such as learned adaptive behaviour, with a change of coping strategy from active to passive (Nishimura et al., 1988; Molendijk and de Kloet, 2015). Because there was no antidepressant treatment involved in the present study, we interpreted the reduction in immobility counts as an increase in active stress coping style (Molendijk and de Kloet, 2015; de Kloet and Molendijk, 2016); importantly, epidemiological studies and systematic reviews indicate that active coping style is a protective factor against depression in humans (Nagase et al., 2009; Labrague, 2021; Alves et al., 2023).

Replicating our previous studies in male adolescents (Ribeiro-Silva et al., 2016; da Silva et al., 2018; Simionato et al., 2022), CSR induced an impairment in body weight gain, and in the present study, this result was extended to females. Whether this deficit is due to increased energy expenditure (Koban and Swinson, 2005; Hipólide et al., 2006; Barf et al., 2012b; Menezes et al., 2020) and/or reduced food intake (Koban et al., 2008; Martins et al., 2008; Barf et al., 2012a; Venancio and Suchecki, 2015) is still a matter of debate. Be that as it may, the fact is that regardless of the protocol used to induce CSR, impaired body weight gain is a consistent finding across several laboratories (Bergmann et al., 1989; Koban and Stewart, 2006; Caron and Stephenson, 2010; Martins et al., 2010; Barf et al., 2012b; Vetrivelan et al., 2012; Venancio and Suchecki, 2015; Murack et al., 2021). Like what has been reported in adult animals (Koban et al., 2008; Venancio and Suchecki, 2015), this result contrasts with reports in human beings. For instance, prolonged exposure to short sleep duration during adolescence is associated with increased body weight (Park and Kim, 2023), especially in girls (Collings, 2022). On the other hand, CSR increased relative adrenal weight in males, again replicating our previous study (da Silva et al., 2018). In that study, relative adrenal weight was determined 1 week after the end of the CSR period, following two behavioural tests. Although females’ adrenals were heavier than those of males, which is expected for this parameter (Miragaia et al., 2018), CSR did not increase them further. Some hypotheses can be raised to explain this result: (1) CSR might have been more stressful to males than to females, since adrenal hypertrophy is an index of chronic stress (Sapolsky et al., 2000); (2) Females habituated to CSR more readily than males; (3) The sleep recovery period was sufficient to restore adrenal weight to CTL values. These hypotheses need to be tested in future studies.

The corticosterone results revealed that the behavioural tests induced different hormonal responses for both males and females and that FST was the test that elicited the highest output. Although in the present study animals were submitted to only one test, based on these data, we propose that, in protocols involving several testing in the same animals, the sequence should be SPT, EPM and FST (from less to more stressful). Although CTL and CSR males secreted similar amounts of corticosterone in the different conditions, CSR females displayed lower corticosterone levels than CTL ones either at baseline or after the behavioural tests. There is evidence from animal studies that active coping reduces HPA axis activity (De Boer et al., 1990; Hsiao et al., 2016), although there is some controversy (Helmreich et al., 2012). In the case of FST, which can be viewed as a stress-coping evaluation (Molendijk and de Kloet, 2015; de Kloet and Molendijk, 2016), the lower corticosterone levels could be explained by the lesser immobility counts in CSR than CTL females.

Protracted sleep deprivation impairs hippocampal-dependent memory and this outcome is closely related to reduction of neurogenesis (for a comprehensive review, see Kreutzmann et al., 2015). Since BDNF is a neurotrophin intrinsically related to neuronal differentiation and survival, there is a high likelihood that sleep deprivation negatively impacts BDNF production. If on one hand there are studies reporting a reduction in BDNF production in sleep-restricted animals of both sexes (Zielinski et al., 2014; Meerlo et al., 2015; Abbasi et al., 2024), on the other hand, many studies report an enhancing effect of total or REM sleep deprivation/restriction on this protein (Fujihara et al., 2003; Wallingford et al., 2014; Maturana et al., 2015). Contrary to our previous study (Machado et al., 2005), no group differences in BDNF concentrations were found in either males or females. This discrepancy may be explained by differences in the testing procedures. Previously, we exposed CTL and CSR males to two behavioural tests in sequence. In the first test (negative sucrose contrast test), animals were maintained individually for 3 days and on the 4^th^ day, they were tested in the EPM and then the brains were collected for biochemical analyses. In the present study BDNF levels were measured in the brains of non-tested animals, to determine whether CSR could influence this parameter. Therefore, it seems that changes in BDNF levels depend on the length and type of method used to induce sleep disruption, but also on the pre-conditions of the animals (tested, stressed, sleep recovered).

Assessment of the phase of the oestrous cycle revealed a similar distribution of the phases in CTL and CSR females, contrary to a previous study showing that 4 days of continuous sleep deprivation had an impact on cyclicity of adult females (Antunes et al., 2006). During the sleep deprivation period, females cycled normally, but for the following 10 days after sleep deprivation, they remained in dioestrus (Antunes et al., 2006). The discrepancy between the present and the previous work can be explained by the type of manipulation and the age of the females. It is possible that the 6 h of free sleep/day was sufficient to recover the hormonal milieu and/or the sleep restriction protocol started before the oestrous cycle was fully developed (pre-pubertal females). Alternatively, in Antunes and colleagues’ work, the oestrous cycle was determined for 14 days, whereas in the present study, a single sample was taken 3 to 4 days after the end of the sleep restriction protocol.

Adolescence is a period of profound physiological and socio-emotional changes (Casey et al., 2015). In humans, difficulties to initiate or maintain sleep emerge during this period of life with greater preponderance in girls than boys. Interestingly, poor sleep quality increases alcohol drinking only in boys, who also show more externalising behaviours than girls, whereas girls display more internalising behaviours than boys (Zhang et al., 2016). Our model showed that during CSR, both males and females exhibited behaviours that emulate cardinal symptoms of depression, e.g., poor self-care and anhedonia. Because sleep plays an important role in stress resilience (Suchecki et al., 2012), a 48-h of free sleep were allowed to all animals after this protocol. In a previous study with adult male rats, this period was sufficient to restore the sleep pattern to pre-sleep restriction levels (Machado et al., 2005). Surprisingly, it seemed that free sleep was more effective for behavioural and physiological recovery in females than in males. Which could be a mediator of this faster or more efficient recovery in females? Based on numerous evidence, we propose that prolactin could be a strong candidate. In previous studies with male rats, we showed that rapid eye movement (REM) sleep deprivation, associated with footshock stress, induced the largest REM sleep rebound and the largest increase in prolactin levels (Machado et al., 2008). On the one hand, prolactin is a hormone associated with REM sleep and classic studies show that stress increases prolactin levels and REM sleep in mice (Meerlo et al., 2001) and rats (Bodosi et al., 2000). Interestingly, this effect seems to be stronger in females (Jefferson et al., 2014). On the other hand, prolactin infusion in the dorsal raphe nucleus increases the amount of REM sleep in a sustained way (Machado et al., 2017). In response to stress, females secrete higher levels of prolactin than males and this phenomenon is observed in mice (Mason et al., 2022), rats (Severino et al., 2004) and humans (Jezová et al., 1996). Thus, based on the evidence presented above, we propose that females secreted higher levels of prolactin, leading to greater REM sleep rebound, which has been claimed to be an important resilience factor against emotional disorders (Suchecki et al., 2012).

This study had a couple of limitations and raised follow-up questions. One limitation is related to attrition that occurred throughout a very long experimental protocol. We faced some technical problems that resulted in the loss of animals, thus reducing the sample size originally planned. The other limitation is the lack of data on the post-CSR rebound that could inform whether there was a sex difference, which could explain the outcomes observed. As a follow-up, one unsolved issue is whether immediately after CSR CORT levels in adolescent rats are elevated, similarly as to what has been reported in adult males (Martins et al., 2010). Another question that remains to be answered is whether in females there is an incubation phenomenon of emotional-related behavioural changes that will manifest in the long-term. In a previous study on the effects of CSR in young mice, some behavioural changes related to sociability were observed 4 weeks after the end of the protocol, but not immediately after (Sare et al., 2016).

In conclusion, our results showed that during the CSR protocol, both males and females displayed depressive-like behaviour. The 48-h sleep recovery appeared to be sufficient to restore normal emotional-related behaviour in females, whereas males showed higher levels of anxiety-like and more passive coping behaviours than their CTL counterparts. Whilst corticosterone response was similar between CTL and CSR males, CSR females displayed a hyporresponsiveness. These results reinforce the need to study both sexes, since there is a clear sex dimorphism in the consequences of disrupted sleep in adolescent rats.

## Data availability statement

The raw data supporting the conclusions of this article will be made available by the authors, without undue reservation.

## Ethics statement

The animal study was approved by Ethics committee of the Universidade Federal de São Paulo (process numbers 9,462,090,218 and 859,090,218). The study was conducted in accordance with the local legislation and institutional requirements.

## Author contributions

AB: Conceptualization, Data curation, Methodology, Writing – original draft. JO: Conceptualization, Data curation, Methodology, Writing – original draft. DS: Conceptualization, Data curation, Formal analysis, Funding acquisition, Investigation, Methodology, Project administration, Resources, Supervision, Validation, Writing – original draft, Writing – review & editing.

## Footnotes

^1^ www.statsoft.com
